# Exceptional epidemics: AIDS still deserves a global response

**DOI:** 10.1186/1744-8603-5-15

**Published:** 2009-11-14

**Authors:** Alan Whiteside, Julia Smith

**Affiliations:** 1 Health Economics Research Division (HEARD), University of KwaZulu-Natal, Westville Campus, Private Bag X54001 Durban, 4001, South Africa; 2 HEARD University of KwaZulu-Natal, Westville Campus Private Bag X54001, Durban, 4001, South Africa

## Abstract

There has been a renewed debate over whether AIDS deserves an exceptional response. We argue that as AIDS is having differentiated impacts depending on the scale of the epidemic, and population groups impacted, and so responses must be tailored accordingly. AIDS is exceptional, but not everywhere. Exceptionalism developed as a Western reaction to a once poorly understood epidemic, but remains relevant in the current multi-dimensional global response. The attack on AIDS exceptionalism has arisen because of the amount of funding targeted to the disease and the belief that AIDS activists prioritize it above other health issues. The strongest detractors of exceptionalism claim that the AIDS response has undermined health systems in developing countries.

We agree that in countries with low prevalence, AIDS should be normalised and treated as a public health issue--but responses must forcefully address human rights and tackle the stigma and discrimination faced by marginalized groups. Similarly, AIDS should be normalized in countries with mid-level prevalence, except when life-long treatment is dependent on outside resources--as is the case with most African countries--because treatment dependency creates unique sustainability challenges. AIDS always requires an exceptional response in countries with high prevalence (over 10 percent). In these settings there is substantial morbidity, filling hospitals and increasing care burdens; and increased mortality, which most visibly reduces life expectancy. The idea that exceptionalism is somehow wrong is an oversimplification. The AIDS response can not be mounted in isolation; it is part of the development agenda. It must be based on human rights principles, and it must aim to improve health and well-being of societies as a whole.

## Introduction

Countries are struggling to deliver on their pledges for universal access to a comprehensive set of interventions for HIV prevention, treatment, care and support, while the global economy is in crisis. At the same time there has been a renewed debate over whether AIDS deserves an exceptional response. This dispute has divided scientists, civil society, researchers and policy-makers. While deliberation is important, we must maintain focus on the 7000 people who are newly infected with HIV every day, and on those who continue to die from a treatable and preventable disease.

The many AIDS epidemics affect countries and specific groups in various ways and to differing degrees. In Swaziland, 26 percent of the adult population is infected, in Kenya 7.1 percent is infected, while in Canada, only 0.4 percent is infected. South Africa, has the highest number of people living with HIV and AIDS in the world - an estimated at 5.7 million men, women and children [1]. AIDS remains the leading cause of death in Africa.

In wealthy countries, localized epidemics occur in specific contexts. On Vancouver's Downtown Eastside, the infection rate amongst commercial sex workers is 26 percent [2]. In Estonia, 72 percent of injecting drug users (IDU) are HIV positive and, while the national rate among IDU in Russia is 14 percent, it is 74 percent in the city of Biysk [3]. In the Ukraine, which is experiencing an already troubling population decline, the World Bank projects that AIDS will cause an additional 300,000-500,000 deaths by 2014 [4]. The HIV prevalence among men who have sex with men in Bangkok and Yangon is estimated at 30 percent [5,6]. AIDS is having differentiated impacts depending on place and population group, and responses must be tailored accordingly. We argue that AIDS is exceptional, but not everywhere. Exceptionalism is defined as the need to recognize that AIDS, in some contexts, presents unique impacts and challenges, and requires a response that is innovative, well resourced and of unprecedented commitment.

## The debate

In the early 1980s, AIDS was a new disease from an unknown retrovirus; its mode of transmission was mysterious, initially affecting mostly the gay population in the West. Its exceptional status was promoted through an alignment of interests of the medical community and gay advocates [7]. There was a real concern that the disease would spread across the populations, which lead to national campaigns with leaflets going to every household in a number of OECD countries (in Britain, remarkably, this campaign 'Don't die of ignorance' took place under a conservative government). When the feared generalised epidemic did not occur in the West, and with treatment becoming available from the mid 1990s, intellectuals called for an end to AIDS exceptionalism [8].

Internationally, AIDS became increasingly 'globalised.' In 2000, the United States National Intelligence Council (NIC) produced the '*The Global Infectious Disease Threat and Its Implications for the United States*' [9]. Six months later, the UN Security Council passed Resolution 1308, stating: "the HIV/AIDS pandemic, if unchecked, may pose a risk to stability and security" [10]. At the 13^th ^International AIDS Conference, in Durban in 2000, the inequity of treatment was highlighted. AIDS had become a chronic disease in the West, and a death sentence elsewhere. Activists demanded that the drugs, which were beyond the reach of most people in the developing world, should be made universally available.

Manufacture and sale of generic drugs, plummeting prices and growing international initiatives, resulted in an astonishing treatment roll-out, making the response once again exceptional. The costs of ART fell from about $10,000 per patient per year to $350 in the early 2000s [11]. In 2002, the Global Fund for AIDS, TB and Malaria was established. In 2003, President Bush pledged $15 billion toward his Presidential Emergency Programme for AIDS Relief (PEPFAR) and the World Health Organization launched the '3 × 5' campaign to get 3 million people on treatment by 2005. Annual funding rose from US $300 million in 1996, to $13.7 billion in 2008 [12].

In our view, AIDS exceptionalism is under attack from two sources. The first were characterized by Stephen Lewis, speaking at the International AIDS Society Conference on Pathogenesis, Treatment and Prevention in Cape Town in July 2009, as: "the pinched bureaucrats and publicity-seeking academics who advocate exchanging the health of some for the health of others - who propose robbing Peter to pay Paul rather than arguing, in principled fashion, that money must be found for every imperative, including maternal and child health, and sexual and reproductive health, and environmental health as well as all the resources required to turn the tide of the AIDS pandemic" [13]. The second group is public health specialists and academics who wish to enter a serious policy debate about health priorities and resources and how they are and should be allocated. They are concerned by what appears in some contexts as disproportionate amounts of funding targeted at AIDS and because of the belief that AIDS activists prioritize it above other health problems. This is a valid dialogue and needs to be entered with honesty and, above all, data.

A series of books and articles set out the exceptionalism debate. Chin argued UNAIDS and AIDS activists perpetuate certain myths about the epidemiology of HIV so as to keep the disease on the political agenda and, by implication, ensure funding and jobs [14]. Pisani wrote that the flow of funds to AIDS "rubs out common sense," and that scientists have allowed themselves to be compromised by the money and politics of the disease [15]. Epstein suggested that the main driver of the epidemic in Africa is concurrent sexual partnering, but that there has been silence on this issue because people, especially male decision-makers, are not prepared to address their own behaviours (or aspirations) [16]. All three allege that the epidemic has been exagerated and money and resources allocated to inappropriate responses.

The strongest (and most polemical) arguments were advanced by England, who claimed that AIDS financing has undermined health systems in developing countries [17,18]. He accuses UNAIDS of creating a vertical program that diverts human and other resources away from general public health priorities and leads to inefficiencies in the public sector. He draws attention to the tendency among some donors to provide large volumes of off-budget funding dedicated to AIDS-specific programs, "which provides no incentives for countries to create sustainable systems, entrenches bad planning and budgeting practices, undermines sensible reforms such as sector-wide approaches and basket funding ... achieves poor value for money, and increases dependency on aid" [19].

The realization that AIDS programs have sometimes, in their single-minded zeal for results in less than optimum contexts, been misinformed and poorly planned should be taken as constructive criticism, instead of eliciting defensive responses. We know funding has not always been applied where it is most required. For example, in West Africa sex trade workers are a core transmitter group, but most prevention funding is applied to the general population. The Commission on AIDS in Asia found that almost 90 percent of all investment in prevention went to areas with insufficient returns [20]. Planning and funding for AIDS programmes must be improved, especially since resources are tight.

There is also the issue of what Peter Piot, former UNAIDS Executive Director, describes as "The health system's myth. The myth that if we just, if we only strengthen health systems this will solve everything, including AIDS" [21]. The impact of AIDS specific initiatives on health systems has been subject of limited empirical research but much debate. Yu, et al. review both sides and conclude that, while there is imperfect data that suggest AIDS programmes occasionally divert resources, the overwhelming evidence indicates that these programmes improve primary care and health outcomes by drawing attention and resources to otherwise ignored regions and populations [22]. Rather than disbanding AIDS programs, the authors argue: "Current scaled-up responses to HIV/AIDS must be maintained and strengthened. Instead of endless debate about the comparative advantages of vertical and horizontal approaches, partners should focus on the best ways for investments in response to HIV to also broadly strengthen the primary health care systems." These conclusions are supported by the analysis of the WHO Maximizing Positive Synergies Collaborative Group [23]. Michel Sidibé, Executive Director of UNAIDS, is applying such findings by maximizing positive externalities of AIDS responses further by seeking opportunities to ensure that they are leveraged to support the Millenuim Development Goals (MDGs), human rights and development agendas more generally- [24] a neccessary response if the universal access targets are to be met in 2010, and the MDGs in 2015.

## Concentrated epidemics: normalize and focus on rights, stigma and discrimination

In October 2008, the Lancet wrote "A view beyond HIV/AIDS will reinforce plurality and justice, protecting minorities and thus wider majorities" [25]. In countries with low prevalence (taken to be generally below three percent), AIDS should be normalised. By normalized we mean that AIDS is viewed and addressed as one of many important health issues that are integrated into public health systems; the disease itself may not be given priority, though the needs of those most at risk and affected may be prioritized. A diverse group of countries fall into this catagory--for example Senegal, India, the Russian Federation, Thailand and Brazil. In these countries the epidemic is concentrated in what are often known as most-at-risk-populations--usually men who have sex with men, injecting drug users and sex workers and their clients. These groups are often stigmatized, marginalized and criminalized, which inhibits the effectiveness of prevention programs and restricts access to public health services. Normalizing the AIDS response in these contexts includes the creation of supportive legal and social environments that enable the provision and uptake of services so everyone benefits from the same rights, treatment and services. Extra measures may need to be taken to ensure that marginalized groups have equal access. It may be that such groups are not seen as meriting special treatment and therefore the role of pressure groups is important.

Addressing AIDS as a 'normal' public health issue counters the stigma that is directed towards at risk groups by labeling them as 'different;' instead of getting 'special treatment' they get the treatment they deserve. This could offset the tendency of some governments to shirk responsibility for providing for these groups by labeling them as 'special interest groups' or those who make specific 'life style choices.'

A 'normal' public health response is also necessarily adaptable; most at risk population groups are not static. For example, in Russia, young male injection drug users were recognized as the most at risk group until recently. However, the proportion of infections amongst women rose from 13.0 percent in 1995 to 44.0 percent in 2006 [26], indicating a developing need for interventions that address women's sexual and reproductive rights, and prevention of vertical transmission. Similarly, the distinction of normalized and exceptional is not static but fluid. For example, in Eastern Europe AIDS could be argued to require an exceptional response as it is contributing to a troubling population decline. In such situations, a public health approach can provide monitoring of infection rates and impacts, and raise the alarm if and when responses need to adapt or scale-up.

## Mid-level prevalence: exceptional responses if aid dependent

Similarly, AIDS should be normalized in countries with mid-level prevalence, except when life-long treatment is dependent on outside resources - as is the case with most African countries - because treatment dependency creates unique sustainability challenges. Once treatment begins, medications must be taken for life. The drugs are expensive and patients will, after a period of time, need to move from first-line (costing about $92 per patient per year) to second-line treatment (at about $1214 per patient per year) [27]. The required expenditure per AIDS patient in sub-Sahara Africa often exceeds per capita health expenditure. For example, in Malawi one programme reported the annual recurrent costs for direct care per patient on ART were $237, [28] while the national health expenditure per capita was $132 per person per year and the government's expenditure was just $14 [29]. Across the border, Mozambique's per capita health expenditure is $9 while Zambia's is $36. These, and countries like them cannot provide treatment without extensive assistance, which will have to be long term and predicable [30]. The poorer the country and the greater the disease burden, the more dependent it will be on international aid to provide treatment and care. Over has argued that this situation creates an 'international entitlement' as foreign nationals become 'entitled' to access to treatment and care financed through aid, and asks if this is sustainable [31].

Between 2005 and 2008, 60 percent of funding for AIDS responses in sub-Sahara Africa came from multilateral, bilateral and philanthropic organizations, and more than half of AIDS funding came from the United States [32]. In Mozambique, 98 percent of funding for HIV and AIDS programmes was provided by international donors, and 78 percent of that from PEPFAR. As Garrett and Schneider write, "Few HIV/AIDS initiatives were designed with the thought of an exit strategy in mind... All too often donor's best intentions to fight HIV/AIDS have increased dependency" [33]. While this situation is troubling, the alternative--interrupting treatment and rupturing the implicit north-south compact of global solidarity--would be even more so. If anything, this extraordinary situation of donor dependency demands new and creative responses that particularly focus on strengthening local public health capacity to provide treatment and care and massively scaling up more effective prevention interventions.

Planning for HIV and AIDS funding also demands urgency: the current economic crisis, which has hit the United States particularly hard, threatens the sustainability of AIDS funding. It is unlikely the required US $25.1 billion for low- and middle-income countries for treatment and prevention programs in 2010 will be forthcoming [34]. The challenge to the international community is now to develop sustainable and innovative financing initiatives to reduce vulnerabilities to fluctuations in international aid.

## High prevalence regions require an exceptional response

Even where treatment is available, AIDS requires an exceptional response in countries with high prevalence (over 10 percent) due to the need to provide treatment or face increased morbidity and mortality, and the continued challenges of implementing effective prevention programs. High incidence of AIDS related illness and death has demographic and social impacts that will be felt for generations. Life expectancy declines, the size and the structure of the populations changes, and numbers of orphans increase. For example, in Botswana life expectancy fell from 56 years during the period 1970 to 1975; to 46.6 years in 2000 to 2005 [35]. In South Africa, the total annual deaths increased by 87 percent from 1997 to 2005, with at least 40 percent estimated to have been AIDS-related. HIV is unique as it spreads predominantly between reproductive age adults, leaving the elderly and young to care for themselves. The number of orphans due to AIDS in sub-Sahara Africa increased from 6,500,000 in 2001, to 11,600,000 in 2007.

In high prevalence countries the epidemic has a particularly unique and troubling characteristic, often referred to as 'the feminisation of AIDS' [36]. In South Africa, women between the ages of 15 and 24 account for 90 percent of new HIV infections. Women are both biologically and socially more vulnerable to HIV; this is often related to women's lack of sexual and reproductive rights. According to the Medical Research Council of Cape Town University, one in four women in South Africa report abuse by an intimate partner [37]. A study from India finds that women who experience intimate partner violence consistently demonstrate greater HIV prevalence [38]. Therefore, Lewis rightly argues, "Bringing an end to sexual violence is a vital component in bringing an end to AIDS" [39]. Though there has been rhetorical commitment to promoting women's rights, we have yet to see outcomes in terms of substantial decreases in levels of gender-based violence and increased sexual and reproductive rights. Too many programs continue to ignore the reality of gender inequality. For example, the popular ABC (abstain, be faithful, use condoms) prevention campaign ignores the reality that wives may not be able to abstain or use condoms without their husband's 'permission.' It does not recognize that in many countries a man cannot be accused, in law, of raping his wife. In Kenya, this contributes to high prevalence rates amongst women aged 15 to 49, which are nearly twice those of men [40]. Addressing the feminization of AIDS requires unprecedented political commitment to and resources for initiatives that promote gender equality. This includes programs that empower men to make choices that are best for their families and communities.

In the context of high levels illness and mortality, the HIV and AIDS response also must recognize the increased care burden placed on women. UNAIDS estimates that 90 percent of HIV and AIDS related caregiving in sub-Sahara Africa is done in the home [41], and the majority of this caregiving is done by women. Research on HIV and AIDS care at the household level suggests it is having disproportionate negative effects on women; it reduces economic and educational opportunities at the same time as increasing household costs, causes emotional strain and poor mental health, and adversely affects women's own physical health [42]. Increased support for caregivers, both financially and politically, can both ensure effective care and treatment programs, and contribute to women's empowerment. A key component of such responses has to be linking caregivers with public health systems and other support structures, while building partnerships and promoting gender equality [43]. This response has to be integrated, and has to be rights-based.

In high prevalence contexts AIDS must be mainstreamed across a nation. For example, in education there will be issues of teacher deaths, children living with HIV and the need to prevention programmes; in agriculture HIV/AIDS may be implicated in lower production [44]. The health sector faces obvious challenges in providing treatment, but frequently ignore the human resource implications of infection among their own staff. Above all politicians and senior civil servants need to recognize that they are faced by a long wave event

We have argued that AIDS is exceptional but that that response should not be mounted in isolation. The Maximizing Positive Synergies report identifies a range of opportunities for building on the results-based programmes established to address HIV and AIDS to strengthen health sectors and systems [45]. For example, the rapid scaling up of interventions to prevent mother-to-child transmission of HIV provide an ideal platform to offer other maternal and child health, as well as sexual and reproductive heath and rights services. HIV service sites can be used for intensified TB case finding, TB therapy and TB infection control. Supply chains developed to deliver AIDS drugs and diagnostics should benefit all drugs and diagnostics--and the same applies to trained staff and surveillance and information systems. Given that more people are infected than put on drugs, and the spiraling costs of treatment, the focus on prevention must not be lost. Prevention and treatment must be implemented hand-in-hand.

## Conclusion

The idea that exceptionalism is somehow wrong is an oversimplification. While normalizing the response where AIDS is located largely in specific population groups can ensure equitable services and treatment by addressing stigma and discrimination. AIDS must be seen as exceptional in those places where it is having long term development impacts due to high incidences of illness and death. Critics of AIDS exceptionalism do not take into account the unique situation where international aid is literally keeping people alive. In high prevalence countries, prevention programs have yet to slow the rate of infection, and finding ways to do so will require creativity and an unprecedented political commitment. The AIDS response can not be mounted in isolation; it is part of the development agenda. It must be based on human rights principles, and it must aim to improve health and well-being of societies as a whole.

## Competing interests

The authors declare that they have no competing interests.

## Authors' contributions

These authors contributed equally to this work.
